# Knowledge of Health Professionals on Folic Acid Use and Their Prescribing Practice in Bahir Dar City Administration, Northwest Ethiopia: Cross-Sectional Study

**DOI:** 10.1371/journal.pone.0170116

**Published:** 2017-01-30

**Authors:** Yeshalem Mulugeta Demilew, Azezu Asres Nigussie

**Affiliations:** 1 School of Public Health, College of Medicine and health Sciences, Bahir Dar University, Bahir Dar, Ethiopia; 2 Midwifery department, College of Medicine and health Sciences, Bahir Dar University, Bahir Dar, Ethiopia; National Institute of Health, ITALY

## Abstract

**Background:**

Taking folic acid supplement during periconception period is effective to prevent neural tube defects. Unfortunately, a minority of Ethiopian women took folic acid supplement during this period. Low consumption of folic acid might be correlated with knowledge and prescribing practice of health professionals. Therefore, this study was conducted to assess knowledge and prescribing practice of health professionals.

**Methods:**

Institution based cross-sectional quantitative study supplemented by qualitative approach using thematic content analysis of in-depth interview was conducted. A total of 424 health professionals were selected by simple random sampling technique. A convenience sampling technique was used to generate the qualitative data. Bivariable and multivariable logistic regression analysis were used to identify factors associated with knowledge of health professionals.

**Result:**

About 47.7% of health professionals had sufficient knowledge and 9.7% of them had prescribed folic acid to women during periconception period. Age, having work experience in ANC clinic, and being a general practitioner were independent predictors for knowledge of health professionals. Lack of guideline to use as a reference, refreshment training and clear direction from health bureau, time constraint, differing patient priorities, and competing topics were some of the reasons for insufficient knowledge and poor practice.

**Conclusion:**

The majority of the health professionals had insufficient knowledge and poor prescribing practice on folic acid during periconception period. Lack of guideline to be used as a reference, refreshment training and clear direction from health bureau, time constraint, differing patient priorities, and competing topics were some of the reasons for low level of knowledge and poor prescribing practice. Thus, guideline to use as a reference, refreshment training, incorporate the topic in the curriculum of health professionals and supportive supervision should be given to increase the knowledge and prescribing practice of health professionals. Regional Health Bureau should give priority on prevention of birth defects.

## Introduction

Provision of folic acid supplement to a women before and during early pregnancy is a gold standard measure to protect neural tube defects (NTDs) and other congenital anomalies[1]. A woman who had no previous pregnancy with NTD can reduce the risk of having a fetus with NTD up to 62% by taking 0.4mg folic acid supplement during the periconception period[1–4]. Women who had previous pregnancy with NTD can also reduce the risk of recurrence up to 72% by taking 4 mg folic acid a day during protective period[5].

Not only this but also, periconception folic acid use is important to prevent several other birth defects such as cardiac, orofacial, limb and renal anomalies[6]. Besides, folic acid consumption lessens pregnancy related complications like small for gestational age, low birth weight, antepartum haemorrhage and perinatal mortality [6–10].

In Ethiopia, folic acid supplement provision during periconception period was very low. Folic acid supplementation has been practiced in tertiary health care facilities where it was given to a woman during protective period. However, in the health care system (such as health posts, health centers, district and zonal hospitals) deemed to be accessible to majority of women, folic acid supplementation is the most forgotten practice[11].

In the health care system immunization and family planning services, antenatal and postnatal cares were given by nurses, midwives and health extension workers. In health centers and health posts, child births were managed by midwives or nurses and health extension workers, respectively. All these contact periods are the opportunities to counsel women about benefit of folic acid. However, majority (76.4%) of Ethiopian women had no awareness on early supplementation benefits and they did not get counseling service on the benefit of folic acid [12]. Public awareness and folic acid consumption during protective period depends on professionals prescribing and counseling practice [13]. Professionals practice in turn, depends on their knowledge and counseling skill [14,15].

In literatures, knowledge and folic acid prescribing practice of health professionals vary from country to country. In United States, universally, health professionals knew the benefit of folic acid and most of them had knowledge on the correct dose and exact time when folic acid is taken to prevent birth defect [10]. Whereas, in developing countries, majority of the health professionals had insufficient knowledge on benefit, correct dose and exact time when folic acid is taken to prevent NTDs [16].

In this country, there was no previous study on knowledge and prescribing practice of health professionals. Therefore, this study was conducted to assess health professionals’ knowledge on periconception folic acid use and their prescribing practice in Bahir Dar city administration. The findings of this study give relevant information on the topic to Amhara National Regional Health Bureau and stake holders to design intervention strategies to promote health professionals knowledge and practice.

## Materials and Methods

### Aim

This study was designed to assess health professionals’ knowledge on periconception folic acid use and their prescribing practice in Bahir Dar city administration.

### Study setting

The study was conducted in Bahir Dar city administration. Bahir Dar city is the capital city of Amhara Regional State, found at 565 km far from and North West of Addis Ababa, Ethiopia. The city administration was classified for administrative purpose into nine urban and nine rural Kebeles and three satellite towns. According to the Amhara national regional state population projection report, in 2015, the total population of the city administration is 297,775, of these, 156, 515 were females. The city administration had three hospitals, ten health centers, four public clinics, two local nongovernmental clinics and 36 private clinics with 15 gynecologists,55general practitioners,68 midwives,352 nurses and 48 health officers who give preventive and curative services[17].

### Study design and study population

Institution based cross-sectional study was conducted from December 1-20/ 2015, using both quantitative and qualitative study design. The qualitative approach used in-depth interview with gynecologists, general practitioners, nurses, midwives and public health professional. While, in quantitative study general practitioners, nurses, health officers and midwives were participated.

### Sample size, sampling procedure and data collection

The sample size was determined using the formula for single population proportion by considering the following assumptions: proportion of knowledge on folic acid use 50%, since there was no previous study, 95% confidence level, 5% of marginal error, and adding 10% of non- response rate. The required sample size was 424. Qualitative data was collected until saturation.

The sample frame (list of health professionals) was obtained from Bahir Dar city administration health office human resource unit. Using this registration logbook, the study participants were selected by simple random sampling technique through lottery method considering proportional to size for each health institution. Participants for in-depth interview were selected using a convenience sampling technique.

Quantitative data were collected using self-administered validated questionnaire. The questionnaire was adopted from other literatures [10],translated and contextualized to the local context. Four diploma nurses and two public health professionals who had not worked in Bahir Dar city administration were recruited as a data collection facilitator and supervisor, respectively. A two days intensive training on the questionnaire and on general approaches to data collection was given for the facilitators and the supervisors. Pre-test was done in similar settings but not included in the main study on 5% of the sample size.

Semi-structured in-depth interviews were conducted with 13 health professionals (2 gynecologists, 2 general practitioners, 3 midwives, 5 nurses and 1 public health professional). After reading information sheet and securing verbal consent, interview was conducted at the participant’s private office. Participants were questioned using standardized face to face semi-structured interviews.

A semi-structured open ended interview guide was developed on the general topic about folic acid. The topic was not addressed in the fixed order, although the first question was about “what can you tell me about folic acid use?” As the interview progressed, issues about the health professionals’ knowledge and prescribing practice of folic acid were addressed. The in-depth interviews were conducted by two MSC public health professionals with previous experience, lasted approximately 50 minutes. Socio-demographic and service related data were collected at the end of the interview.

Knowledge of health professionals’ on folic acid use was assessed by eight item questions which had yes or no response. Health professionals who had five and above were leveled as had “sufficient knowledge”, whereas, those who had below five were leveled as had “insufficient knowledge”.

Good prescribing practice-in this study, health professionals who gave counseling and supplement or treatment dose of folic acid to a woman at least one month before and three months after pregnancy had good prescribing practice. Whereas, professionals who did not gave counseling service and supplement or treatment dose of folic acid to a woman during protective period had poor prescribing practice.

Periconception period (protective period)- is a period at least one month before and three months after pregnancy.

In Ethiopia, health officers are health professionals who trained at least four years at higher institutions and can deliver clinical and preventive health services at health care institution.

### Data quality control

To assure the quality of data training was given for facilitators and supervisors, pretest was done, standardized questionnaire and interview guide was used. Each day collected information was reviewed and errors were returned to facilitators for correction. Supervisors and investigators closely supervised data collection technique.

### Data processing and analysis

Data were entered and analyzed using SPSS version 20 soft ware. Descriptive statistics were used to describe data. Bivariable and multivariable logistic regression were employed to identify factors associated with knowledge of professionals. P value ≤ 0.2 was taken as a cut-off point to select variables eligible for multiple logistic regression models. P-values ≤ 0.05 was considered statistical significant.

All in-depth interviews were conducted in the Amharic language and tape recorded. The recording of the interviews transcribed verbatim and translated in to English. The data analyzed by using thematic analysis[18–20]. The qualitative analysis was started with individual reading by researchers. The analysis was continued during data collection and the encoding process using the constant comparative method, which consists of analyzing the interviews by comparing one response to earlier reported responses[21]. This analysis was subsequently used to established analytical categories that used as bases of final grid used by the researchers to analyze the transcripts in order to maximize theoretical sensitivity and rigour[19,22]. In accordance to the objective of the study, the analysis was focused on the identification of key elements on knowledge and prescribing practices and similarities and differences. Finally, background data was used to allow comparisons. The interview data were triangulated with the quantitative data.

### Ethical clearance

The study was approved by Ethical review committee of College of Medicine and Health Science, Bahir Dar University. The purpose of the study, rights of participants, potential risk and benefits was explained. Verbal consent was secured from each participant. The confidentiality was maintained throughout the study by excluding personal identifiers from the data collection form.

## Results

### Socio-demographic characteristics of health professionals

Among a total of 424 participants sampled, 392 of them participated, making the response rate of 92.4%. The mean (±SD) age of the respondents was 30.55 (±7.42) years. Nearly two third, 252 (64.3%) participants were females. Almost all, 374 (95.4%) respondents were from Amhara ethnic group. Majority, 360 (91.8%) participants were Orthodox Christian followers. Two hundred fifty five (65.1%) respondents were nurses in their profession (Table 1).

**Table 1:** Socio-demographic characteristics of the health professionals in Bahir Dar city administration, Northwest Ethiopia, December 1-20/ 2015.

Variable	Frequency (n = 392)	Percentage
**Age(in years)**		
**20–24**	48	12.3
**25–29**	184	46.9
**30–34**	66	16.8
**35–39**	42	10.7
**±40**	52	13.3
**Sex**		
**Female**	252	64.3
**Male**	140	35.7
**Ethnicity**		
**Amhara**	374	95.4
**Agew**	13	3.3
**Guragea and Tigray**	5	1.3
**Religion**		
**Orthodox**	360	91.8
**Muslim**	16	4.1
**Protestant**	14	3.6
**Adventist**	2	0.5
**Professional category**		
**Nurses**	255	65.1
**Mid wives**	59	15.0
**General practitioners**	47	12.0
**Health officers**	31	7.9
**Institution they had been worked**		
**Public**	296	75.5
**Private**	81	20.7
**Local NGO**	15	3.8

### Service related information of health professionals

One hundred seventy (43.4%) respondents had less than 5 years work experience. Forty five (11.5%) health professionals took in service training about vitamin supplementation. About 252 (64.3%) and 250(63.8%) participants had worked in ANC clinic and in labor and delivery ward, respectively. One in three, 128 (32.7%) participants attended birth of a neonate with NTDs (Table 2).

**Table 2:** Service related information of health professionals in Bahir Dar city administration, Northwest Ethiopia, December 1-20/ 2015.

Variable	Frequency (n = 392)	Percentage
**Work experience (in years)**		
**<5**	170	43.4
**5–9**	120	30.6
**≥10**	102	26.0
**Have been worked in ANC clinic**		
**Yes**	252	64.3
**No**	140	35.7
**Have been worked in labor and delivery ward**		
**Yes**	250	63.8
**No**	142	36.2
**Got in service training on vitamin supplement**		
**Yes**	45	11.5
**No**	347	88.5
**Attended birth of a neonate with NTDs**		
**Yes**	128	32.7
**No**	264	67.3

### Knowledge of health professionals on folic acid use

Two in three, 258(65.8%) professionals identified at least one food source of folic acid and220 (56.1%) of them knew the benefit of folic acid to prevent NTDs. However, 174 (44.4%) participants had no knowledge on the benefit of folic acid to prevent NTDs. One in five, 86 (21.9%) health professionals knew the correct time when folic acid is taken to reduce the risk of birth defect. Only,20 (5.1%) respondents knew the correct dose of folic acid needed to a woman who has no previous pregnancy affected with NTD, and 10 (2.5%) participants reported the amount of folic acid needed to prevent recurrence. Overall, 187(47.7%) health professionals had sufficient knowledge on periconception folic acid use (Table 3). General practitioners (95.7%) were more knowledgeable than other health professionals, followed by health officers (64.5%) and midwives (62.7%) but nurses (33.3%) were less knowledgeable than other health professionals (Table 3).

**Table 3:** Knowledge of Professionals on periconception folic acid use in Bahir Dar city administration, Northwest Ethiopia, December 1-20/ 2015.

Variable	Frequency (n = 392)	Percentage
**Folate can be found from food staffs**		
Yes	258	65.8
No	134	34.2
Folic acid deficiency causes NTDs		
Yes	218	55.6
No	174	44.4
Folic acid used to prevent NTDs		
Yes	220	56.1
No	172	43.9
NTDs are congenital abnormality		
Yes	259	66.1
No	133	33.9
Pregnant and non pregnant women are targets to folic acid supplement to decrease risk of NTDs		
Yes	106	27.0
No	286	73.0
To prevent NTDs, folic acid supplement should be taken during Periconception period		
Yes	86	21.9
No	306	78.1
Correct dose of folic acid for a women had no previous pregnancy with NTD is 0.4mg		
Yes	20	5.1
No	372	94.9
Correct dose of folic acid for a women with a prior NTD affected pregnancy is 4mg		
Yes	10	2.5
No	382	97.5
Over all knowledge		
Sufficient knowledge	187	47.7
Insufficient knowledge	205	52.3

Similarly, from the in-depth interview, gynecologists and general practitioners had better knowledge than other health professionals. They had knowledge on food source and benefit of folic acid, correct dose and exact time when folic acid is taken to reduce the risk of birth defect. A 43 years old gynecologist with more than 15 years work experience said that *“NTDs are common birth defects in Ethiopia, I personally, gave care for many pregnancies affected by NTD but the problem was not get attention by the government and health professionals. We can prevent NTDs by give folic acid supplement to a woman, if a woman takes folic acid at least one month before and 3 months after pregnancy, the risk of NTDs can reduced up to 70%. A woman who has no previous pregnancy affected with NTD needs a supplement dose of folic acid (0.4mg) orally per day. Whereas, risk woman (a woman who had previous pregnancy affected by NTD, family history, diabetes mellitus and a woman who have taking anticonvulsant medication) needs a treatment dose of folic acid (4 mg) orally per day during protective period.”*

From the three midwives interviewed, only one had knowledge on the benefit, protective period and exact dose of folic acid needed to prevent birth defects. A 25 years old BSC midwife with 2 years work experience said that *“……we can prevent NTDs by give folic acid supplement to all reproductive age women during protective period. Less than 4mg folic acid orally per day is enough for a woman who has no previous pregnancy affected with NTD. Whereas, for the prevention of recurrence of NTD a woman should be take 4mg folic acid during protective period.”*

Nevertheless, nurses were less knowledgeable compared with other health professionals. From the five nurses interviewed, only one nurse correctly identified the benefit and exact time when folic acid is taken to prevent NTDs. Surprisingly, no one had knowledge on exact dose of folic acid needed to prevent birth defects. A 32 years old BSC nurse with 18 years work experience said that *“ ----The recommended amount of folic acid is one tab orally per day 2 months before and 3 months after pregnancy but I don’t know exact dose in gram or milligram.”*

### Folic acid prescribing practice of health professionals during periconception period

About 38(9.7%) health professionals had prescribed folic acid to women during periconception period. Of them, 30(78.9%) were general practitioners. Nevertheless, only 12(31.6%) respondents had prescribed the right dose of folic acid for a woman to prevent recurrence of NTD. However, no one gave folic acid supplement for a woman with no previous pregnancy with NTD during protective period (Table 4).

**Table 4:** Folic acid prescribing practice of health professionals during periconception period in Bahir Dar city administration, Northwest Ethiopia, December 1-20/ 2015.

Variable	Frequency (n = 392)	Percentage
**Gave folic acid to a woman during periconception period**		
**Yes**	38	9.7
**No**	354	90.3
**Counseled a woman on benefit of folic acid**		
**Yes**	38	9.7
**No**	354	90.3
**Prescribed 4mg folic acid for risk mother (n = 38)**		
**Yes**	12	31.6
**No**	26	68.4

From the in-depth interview report, in tertiary hospitals, where gynecologists and general practitioners give care for the women, folic acid was given for the women to prevent recurrence of birth defects. A 28 years old general practitioner with 1year and 9months work experience said that

“*………in this hospital folic acid supplement is given for the women with previous pregnancy affected with NTD*. *From my recent experience*, *today*, *a woman who had previous history of giving birth with spina bifida; gives birth of a normal baby after taking folic acid during periconception period*. *She again counseled to take folic acid before and during her next pregnancy*. *In general*, *after counseling*, *4mg folic acid is given to all women who gave birth of a neonate affected with NTD*. *However*, *due to high work load and negligence folic acid was not prescribed to* a woman with no previous pregnancy with NTD.*”*

In the health centers and clinics, where nurses and midwives give care for the women; folic acid supplement was not given to the women during periconception period. A 24 years old diploma midwife with 2 years and 5 months work experience explained that

“……*in this health center folic acid was not given to the women during protective period. Recently iron with folic acid is given for all pregnant women who come to get antenatal care service to prevent anemia. But, majority of pregnant women came to get antenatal care after sixteen weeks of gestation and no opportunity to give folic acid during periconception period. During family planning service provision has no one go further to take history about their plan to have pregnancy to counsel and give folic acid during protective period. Lack of guideline to use as a reference like anemia prevention guideline is the main reason to not prescribe folic acid during protective period. Though, we have the awareness about the advantage of folic acid supplementation during the periconception period, we need to have a clear direction from Regional Health Bureau or it shall be prepared as a guideline to use as a reference.”*

Another 28 years old BSC nurse with 6 years work experience expressed that *“in this health center folic acid was not given to the women during protective period*. *As to me*, *I don’t know the benefit of folic acid supplement to prevent NTDs because its importance is not incorporated in the curriculum and teachers had not taught it during my University stay.”*

### Factors associated with knowledge of health professionals

In the bivariable logistic regression analysis sex, having work experience in ANC clinic, having work experience in labor and delivery ward, assisting a woman who gave birth of a neonate with NTD, service year, age, professional category and type of health institution where they had worked showed statistically significant association with knowledge of the health professionals (Table 5).

**Table 5:** Factors associated with knowledge of health professionals on periconception folic acid use in Bahir Dar city administration, Northwest Ethiopia, December 1-20/ 2015.

Factor	Knowledge		COR(95%C/I)	AOR(95% C/I)
	Sufficient	Insufficient		
**Sex**				
**Male**	79(20.1)	61(15.6)	1.7(1.13,2.62)	
**Female**	108(27.6)	144(36.7)	1.00	
**Working in ANC clinic**				
**Yes**	142(36.2)	110(28.1)	2.7(1.76,4.20)	1.9(1.09,3.53)*
**No**	45(11.5)	95(24.2)	1.00	1.00
**Working in labor and delivery ward**				
**Yes**	138(35.2)	112(28.6)	2.3(1.52,3.52)	
**No**	49(12.5)	93(23.7)	1.00	
**Assist a women who gave birth of a neonate with NTD**				
**Yes**	76(19.4)	52(13.3)	2.0(1.31,3.09)	
**No**	111(28.3)	153(39.0)	1.00	
**Service year (years)**				
**<5**	112(28.6)	100(25.5)	1.5(1.05,2.34)	
**≥5**	75(19.1)	105(26.8)	1.00	
**Age (years)**				
**≤30**	126(32.1)	106(27.0)	1.9(1.28,2.90)	1.7(1.00,2.89*)
**>30**	61(15.6)	99(25.3)	1.00	1.00
**Professional category**				
**General practitioners**	45(11.5)	2(0.5)	38.7(9.22,163.15)	34(7.57,160.67)*
**Midwives**	37(9.4)	22(5.6)	2.8(1.62,5.17)	2.0(1.05,4.01)*
**BSC nurses and health officers**	105(26.8)	181(46.2)	1.00	1.00
**Institution have been worked**				
**public**	149(38.0)	147(37.5)	1.54(0.96,2.47)	
**private**	38(9.7)	58(14.8)	1.00	

AOR = Adjusted Odds Ratio; COR = Crude Odds Ratio; **95%**CI = ninety five percent confidence interval,

* = P-value <0.05

In the multivariable logistic regression analyses, professionals who had work experience in ANC clinic [AOR = 1.9, 95% CI: (1.09, 3.53)], age less than 30years old [AOR = 1.7, 95% CI: (1.00, 2.89)] and professional category; being a general practitioners [AOR = 34.0, 95% CI: (7.57, 160.67)] and midwives [AOR = 2.0, 95% CI: (1.05, 4.01)] had significant association with knowledge of the health professionals on periconception folic acid use (Table 5).

## Discussion

Although, many pregnancies were affected by NTDs; in Ethiopia, the prevention method was not well implemented. In this study, less than half (47.7%) of health professionals had sufficient knowledge on periconception folic acid use. Low level of knowledge was also reported from the in-depth interview, particularly among nurses and midwives. This finding was consistent with the study findings in developing countries [16,23,24]. Low level of knowledge might be due to lack of refreshment training and guidelines on the topic. Additionally, majority of the participants have not been worked in ANC clinic, labor and delivery ward, so they were less likely to update their knowledge about congenital abnormalities and its preventive methods.

Nevertheless, this finding was lower than the study finding in Florida (58%)[10]. This discrepancy might be due to difference between study settings. This study was done in developing country, in which the curriculum gives emphasis for communicable disease than congenital abnormalities. Whereas, the study in Florida was done in developed world, in which congenital abnormalities get better attention.

About 56.1% of health professionals had knowledge on the benefit of folic acid and 21.9% of respondents had knowledge on the recommended period in which folic acid is prescribed to prevent birth defect. Only, 5.1% and 2.5% of respondents recognized the occurrence and recurrence prevention dose of folic acid, respectively. This finding was lower than previous study findings [23,25,26]. This might be due to difference between study settings and study subjects; the curriculum, refreshment training and directions on service provision are different between the settings.

Only few, 9.7% of respondents counsel and gave folic acid supplement to pregnant women during periconception period. Similar finding was also reported from the in-depth interview. This finding was lower than the study findings in Iran and Canada[16,27]. Only 12 participants recommend the right dose of folic acid during protective period to prevent recurrence of birth defect. This result was lower than the study findings in Israel and Ohio[26,28]. This discrepancy might be due to difference between the study settings and study subjects. This study includes gynecologists, physicians, midwives, health officers and nurses with different level of education whereas the former studies included only gynecologists and physicians with better educational status.

Surprisingly, in this study, only, gynecologists and general practitioners prescribed folic acid during periconception period. This might be due to difference in curriculum and course content they took during their University stay. Doctors took course on congenital abnormality and their preventive methods in detail than other professionals. Additionally, Doctors had long clinical attachment period than other professionals which creates a good opportunity to attend birth with NTD and lesson on prevention methods. These experiences could lead them to explore more on the topic. Therefore they are more likely to prescribe folic acid during protective period.

Significant association was observed between having work experience in ANC clinic and professionals knowledge on periconception folic acid use. Health professionals who had worked in ANC clinic were 1.9 times more likely to had sufficient knowledge than those who did not work in ANC clinic. This finding was consistent with previous study finding[10]. This might be due to the reason that, professionals who worked in ANC clinic were more likely to get update training on MCH service including prevention of congenital abnormality. Additionally, those who worked in ANC clinic were more likely to explore information on supplementation because this is one component of ANC care.

Age was one determinant factor to knowledge of health professionals on periconception folic acid use. In this study, professionals whose age less than 30years old were 1.7 times more likely to had sufficient knowledge than their counter part. This might be due to the fact that younger health professionals had attended their education recently and they had motivation to learn up to date information.

Professional category had significant association with their knowledge level on periconception folic acid use; general practitioners and midwives had better knowledge than nurses. General practitioners were 34 times and midwives were 2 times more likely to have sufficient knowledge compared to nurses or health officers. Similar finding was also reported from the in-depth interview. This finding was in agreement with the study findings in Israel and United States [10,28]. This might be due to difference in the curriculum, doctors and midwives took courses on congenital abnormality and their protective methods in detail than other health professionals. Moreover, most commonly, physicians and midwives work in ANC clinics and delivery wards, so, they may had experience to assist delivery with birth defect and they explored on the preventive methods.

From the in-depth interview, reasons for low level of knowledge and poor prescribing practice were time constraint, differing patient priorities, competing topics, lack of clear direction from Health Bureau, lack of refreshment training and guidelines. Similar finding was reported from previous study finding[29].

In this study some confidence intervals were somehow wide due to low number of cases per variables which would affect the precision of estimates. This study is also limited to shortage of reference material.

## Conclusion

The majority of the health professionals had insufficient knowledge and poor prescribing practice on folic acid during periconception period. Factors associated with sufficient knowledge of health professionals were being a general practitioner, having work experience in ANC clinic and age less than 30years old. Lack of guideline to use as a reference, refreshment training and clear direction from health bureau, time constraint, differing patient priorities, and competing topics were some of the reasons for low level of knowledge and poor prescribing practice.

## Recommendation

Thus, guideline to use as a reference, refreshment training, incorporated the topic in the curriculum of health professionals and supportive supervision should be given to increase the knowledge and prescribing practice of health professionals. Regional Health Bureau should give priority on prevention of birth defects.

## Supporting Information

S1 Dataset The SPSS data, which is the original data to this manuscript, is attached as supportive data. (SAV)

## Abbreviations

ANC: Antenatal Care
IUFD: Intrauterine Fetal Death
NGO: Nongovernmental Organization
NTD: Neural Tube Defect
